# Neonatal Renal Vein Thrombosis

**DOI:** 10.7759/cureus.113967

**Published:** 2026-08-04

**Authors:** Celine Mayeko-Coklee, Tsolaye Ugbeye, Martijn Tilanus

**Affiliations:** 1 Emergency Medicine, Sinai Chi, Chicago, USA; 2 Surgery, American University of the Caribbean, Cupecoy, SXM; 3 Pediatrics, Sint Maarten Medical Center, Philipsburg, SXM

**Keywords:** hematology, internal medicine, neonatal renal vein thrombosis, paediatric emergency medicine, paediatrics, protein c deficiency, renal vein thrombosis (rvt), rvt

## Abstract

Neonatal renal vein thrombosis (NRVT) is a rare and potentially fatal condition that can be frequently misdiagnosed as an infection by clinicians due to its nonspecific presentation. This report presents a preterm neonate who developed a renal vein thrombosis (RVT) complicated by adrenal hemorrhage as the result of an underlying protein C deficiency. This case highlights the importance of maintaining a broad differential when treating seemingly inconspicuous symptoms and identifying underlying etiologies. Increased understanding of this topic is essential for early diagnosis, management, and improved clinical outcomes for affected patients.

## Introduction

Neonatal renal vein thrombosis (NRVT) is a condition in which a blood clot forms in one or both renal veins during the newborn period, typically presenting antenatally and up to one month after birth [1].It is a rare and potentially fatal condition that can be frequently misdiagnosed as an infection by clinicians due to its nonspecific presentation. The condition is frequently associated with risk factors such as prematurity, dehydration, sepsis, indwelling vascular catheters, and inherited thrombophilias, including protein C deficiency [2,3]. Because its clinical presentation can be variable and nonspecific, diagnosis may be delayed, particularly when patients present with atypical findings. Delayed diagnosis and treatment are often associated with long-term complications associated with significant morbidity, including renal atrophy, chronic kidney disease, hypertension, and permanent loss of renal function. 

We report the case of a preterm female neonate who presented with unilateral lower extremity swelling and discoloration and was subsequently diagnosed with left renal vein thrombosis (RVT) with extension into the inferior vena cava (IVC), adrenal hemorrhage, and underlying protein C deficiency. This case aims to highlight the importance of maintaining a broad differential when evaluating pediatric patients with unusual limb findings, exploring underlying etiologies, and emphasizing the role of timely imaging and thrombophilia evaluation in guiding diagnosis and management.

## Case presentation

**Figure 1:**
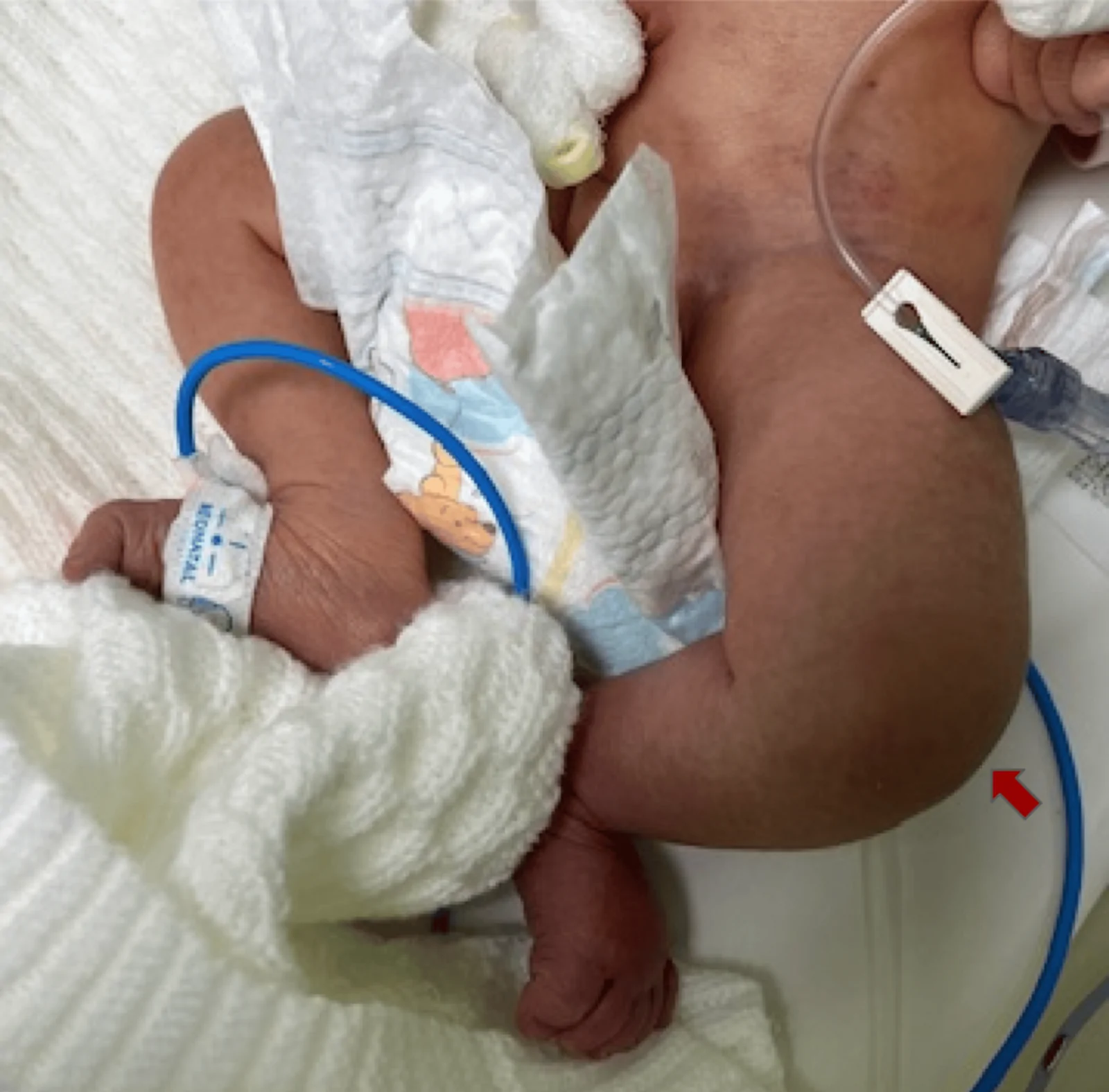
Localized swelling observed in the left lower extremity (red arrow), specifically involving the ankle and foot

A female neonate was first examined by a pediatrician on the first day after birth. On initial physical examination, she appeared slightly icteric. Localized swelling was observed in the left lower extremity, specifically involving the ankle and foot (Figure 1). A small erythematous spot was noted on the medial aspect of the left ankle, and the leg appeared slightly darker than the rest of the body. Despite these findings, the left leg maintained normal temperature and inguinal pulsation.

Over the next day, the swelling progressed proximally to involve the entire left leg, extending from the inguinal region. The limb developed a bluish-purple discoloration and became tender to touch. The absence of erythema and warmth made an infectious cause less likely.

**Figure 2:**
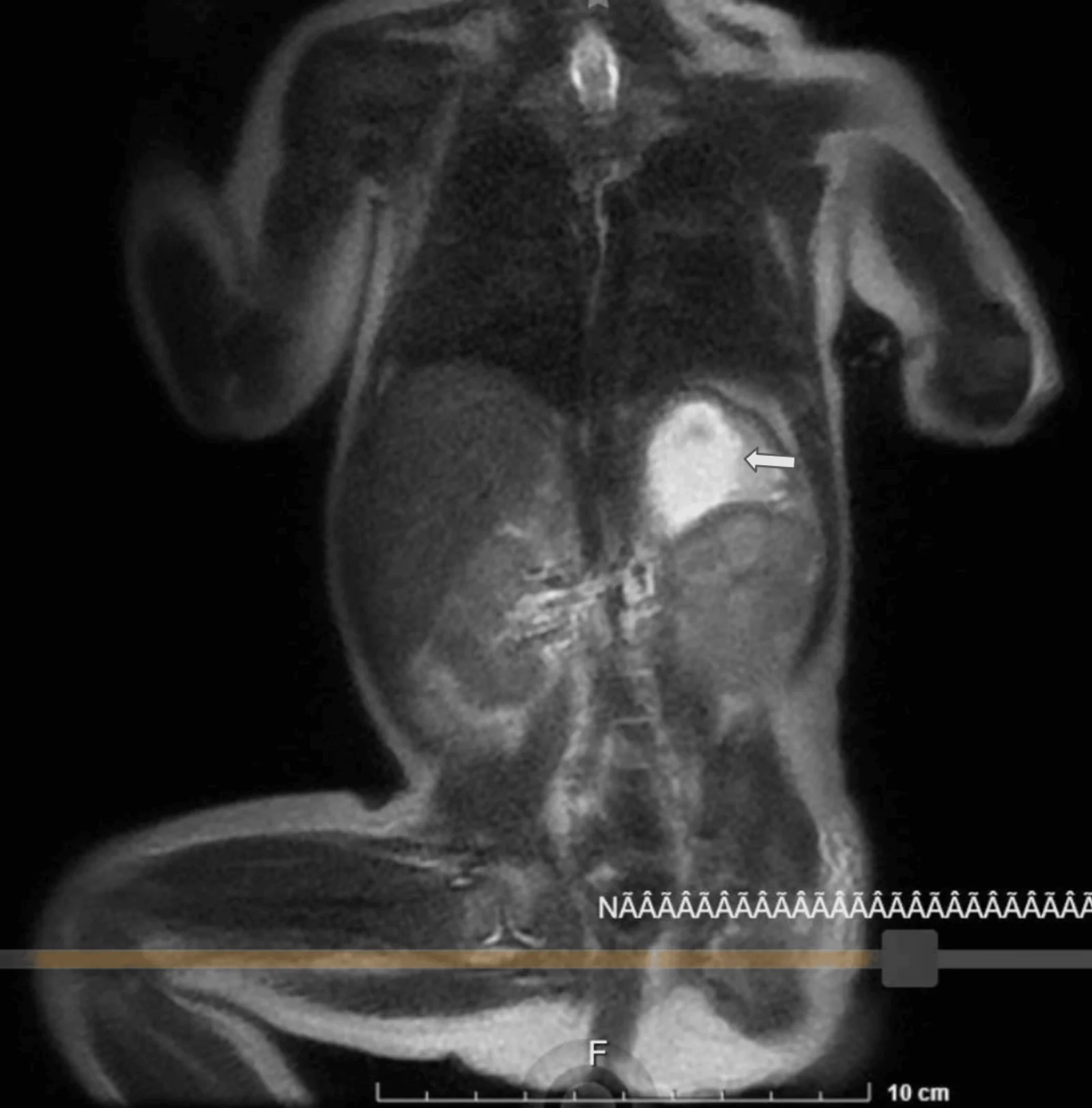
Left adrenal hemorrhage visualized on MRI as a 3.6 cm × 2.5 cm triangular shaped mass

Ultrasound of the abdomen and left inguinal region was performed, revealing no evidence of deep vein thrombosis (DVT) or arterial stenosis. However, a space-occupying lesion was identified in the region of the left adrenal gland. Subsequent MRI of the abdomen confirmed a 3.6 cm × 2.5 cm mass at the upper pole of the left kidney (Figure 2). Differential diagnoses included adrenal hemorrhage, adrenal tumor, and neuroblastoma.

Laboratory studies ruled out infection, with normal WBC counts and decreasing C-reactive protein (CRP) levels. Concurrently, the patient exhibited worsening jaundice, for which phototherapy was initiated, resulting in significant improvement.

In the following days, the swelling of the left leg decreased, though fullness persisted in the inguinal region. Based on the evolving clinical picture and imaging findings, a diagnosis of left renal vein thrombosis (RVT), IVC thrombosis, and left adrenal hemorrhage was made, along with associated neonatal hypertension. The patient was referred to the neonatal intensive care unit (NICU) and initiated on subcutaneous nadroparin. Due to the need for ongoing monitoring of anti-Factor Xa levels and specialized care, the patient was transferred to a tertiary center.

At the referral hospital, further investigations were conducted, including chest and abdominal CT imaging, anti-Xa monitoring, thrombophilia screening (Factor V Leiden, prothrombin gene mutation), and hemoglobin electrophoresis. A previously obtained test had revealed protein C deficiency (33%; normal: 70%-140%), which was communicated to the managing team.

Anticoagulation therapy continued with a 72-hour course of heparin to attempt resolution of the IVC thrombus. Imaging showed persistent chronic thrombi. The medical team concluded that the thrombus posed no immediate threat, and anticoagulation was transitioned to enoxaparin. Follow-up imaging demonstrated partial resolution of the IVC thrombus and partial restoration of venous flow. The left adrenal gland also returned to normal size on subsequent evaluation.

## Discussion

Management

Although the treatment for NRVT has varied over the past two decades, the current standard of care is guided by the location of the thrombus and the degree of renal impairment. For patients with unilateral RVT, conservative management with symptomatic support is favored. For those presenting with unilateral RVT extending into the IVC and for bilateral RVT, antithrombotic therapy may be attempted [4].

In the case of this infant with a thrombus involving the left renal vein with extension into the IVC and normal renal function, the standard of care involves initiating and continuing anticoagulant therapy for six weeks to three months depending on the infant’s clinical needs and response to treatment [5]. Low molecular weight heparin (LMWH) is the drug of choice due to its high bioavailability when administered via subcutaneous injection [6]. This is an important consideration, as poor venous access in newborns may make other forms of therapy difficult. The use of LMWH has proven effective, with 94% of neonates demonstrating thrombus resolution [7]. Treatment should be monitored and adjusted according to anti-factor Xa levels to prevent adverse effects such as bleeding, which can occur in 3.9% of neonates younger than 28 days of age [8]. Other adverse effects include heparin-induced thrombocytopenia and osteoporosis, both of which are rare and occur less frequently with LMWH than with unfractionated heparin (UFH) [9].

When initiating anticoagulant therapy in neonates, it is important to recognize that they often require higher weight-based dosing and may take longer to achieve therapeutic levels compared with older children and adults. This is likely due to decreased thrombin generation and increased renal clearance in children [10].

In select cases, thrombolytic therapy may be considered; however, several contraindications must be evaluated before treatment initiation. These include major surgery or hemorrhage within the previous 10 days, neurosurgery within the previous three weeks, severe asphyxial events within seven days, invasive procedures within three days, seizures within 48 hours, prematurity (<32 weeks gestation), septicemia, active bleeding, and the inability to maintain platelet counts greater than 100,000/μL or fibrinogen levels greater than 100 mg/dL (>1 g/L) [11]. These factors should be carefully evaluated by an interdisciplinary team before initiating therapy.

Long-term management of pediatric patients with severe or homozygous protein C deficiency-related thrombosis includes the use of direct-acting anticoagulants (DOACs). Treatment often begins with heparin or LMWH, eventually switching to warfarin with frequent monitoring of the INR [12]. With appropriate follow-up and management, many patients can achieve favorable long-term outcomes, going on to live relatively normal lives with minimized risk of recurrent thrombosis.

Etiology, clinical presentation, and diagnosis

Neonatal renal vein thrombosis is a rare but serious condition that may occur secondary to maternal risk factors, hypercoagulable states in the mother and/or infant, or medical interventions such as intravascular catheter placement [2]. In this case, the thrombosis was attributed to an underlying protein C deficiency identified postnatally. Protein C deficiency is characterized by a partial or complete absence of protein C, a vitamin K-dependent plasma serine protease involved in the regulation of blood coagulation [3]. This deficiency occurs in approximately one in 500 individuals and is inherited in an autosomal dominant manner, with disease severity varying depending on whether the affected individual is homozygous or heterozygous for the PROC gene mutation [3]. Clinical manifestations range from asymptomatic disease to severe thrombosis that may occur prenatally and is associated with significant morbidity and mortality.

The classic presentation of NRVT includes hematuria, thrombocytopenia, and a palpable abdominal mass secondary to renal enlargement. However, these signs and symptoms may be masked in critically ill neonates, making diagnosis challenging [13]. Diagnosis is established through laboratory investigations and imaging studies.

Because spontaneous RVT is uncommon in neonates, it is essential to evaluate for underlying etiologies in these patients. Common predisposing conditions include prematurity, dehydration, sepsis, birth asphyxia, shock, maternal diabetes, polycythemia, cyanotic congenital heart disease, and iatrogenic causes, particularly the presence of indwelling umbilical venous catheters [13]. Hematologic disorders associated with hypercoagulability, including inherited thrombophilias such as protein C deficiency, may also contribute to the development of RVT [14].

Prognosis and long-term implications

The literature surrounding RVT in neonates and pediatric patients remains limited. However, available studies suggest that children with a history of RVT, regardless of age at presentation, are at increased risk of developing chronic kidney disease (CKD) and hypertension later in life [13]. Prognosis for these patients is influenced by the timing of diagnosis and the prompt initiation of treatment. Therefore, pediatric patients with a history of RVT should undergo routine long-term monitoring for the development of CKD and/or hypertension.

## Conclusions

This case highlights an uncommon presentation of NRVT with extension into the IVC, initially presenting with unilateral lower-extremity swelling and discoloration. The diagnosis was ultimately established through imaging and thrombophilia evaluation, which identified an underlying protein C deficiency as a significant contributing factor. Early recognition of RVT and its underlying etiologies is essential for guiding appropriate anticoagulant therapy and optimizing clinical outcomes. This case underscores the importance of maintaining a broad differential diagnosis in neonates with atypical limb findings and supports the role of timely diagnostic evaluation and multidisciplinary follow-up in the management of neonatal thrombotic disease.
